# Venom of the Coral Snake *Micrurus clarki*: Proteomic Profile, Toxicity, Immunological Cross-Neutralization, and Characterization of a Three-Finger Toxin

**DOI:** 10.3390/toxins8050138

**Published:** 2016-05-05

**Authors:** Bruno Lomonte, Mahmood Sasa, Paola Rey-Suárez, Wendy Bryan, José María Gutiérrez

**Affiliations:** 1 Instituto Clodomiro Picado, Facultad de Microbiología, Universidad de Costa Rica, San José 11501, Costa Rica; msasamarin@gmail.com (M.S.); wenjbq@gmail.com (W.B.); jose.gutierrez@ucr.ac.cr (J.M.G.); 2 Programa de Ofidismo y Escorpionismo, Universidad de Antioquia, Medellín 050010, Colombia; ofidpa@gmail.com

**Keywords:** monadal coral snake, venom, toxin, *Micrurus*, antivenom, proteome, Elapidae, neutralization

## Abstract

*Micrurus clarki* is an uncommon coral snake distributed from the Southeastern Pacific of Costa Rica to Western Colombia, for which no information on its venom could be found in the literature. Using a ‘venomics’ approach, proteins of at least nine families were identified, with a moderate predominance of three-finger toxins (3FTx; 48.2%) over phospholipase A_2_ (PLA_2_; 36.5%). Comparison of this venom profile with those of other *Micrurus* species suggests that it may represent a more balanced, ‘intermediate’ type within the dichotomy between 3FTx- and PLA_2_-predominant venoms. *M. clarki* venom was strongly cross-recognized and, accordingly, efficiently neutralized by an equine therapeutic antivenom against *M. nigrocinctus*, revealing their high antigenic similarity. Lethal activity for mice could be reproduced by a PLA_2_ venom fraction, but, unexpectedly, not by fractions corresponding to 3FTxs. The most abundant venom component, hereby named clarkitoxin-I, was identified as a short-chain (type I) 3FTx, devoid of lethal effect in mice, whose target remains to be defined. Its amino acid sequence of 66 residues shows high similarity with predicted sequences of venom gland transcripts described for *M. fulvius*, *M. browni*, and *M. diastema*.

## 1. Introduction

Coral snakes (*Elapidae*) comprise some 71 species that are widely distributed in tropical and subtropical regions of the New World, from Southeastern USA to Central Argentina [1,2,3]. Envenomings by these snakes are far less frequent than those inflicted by viperids, but are of medical concern [4,5].

**Figure 1:**
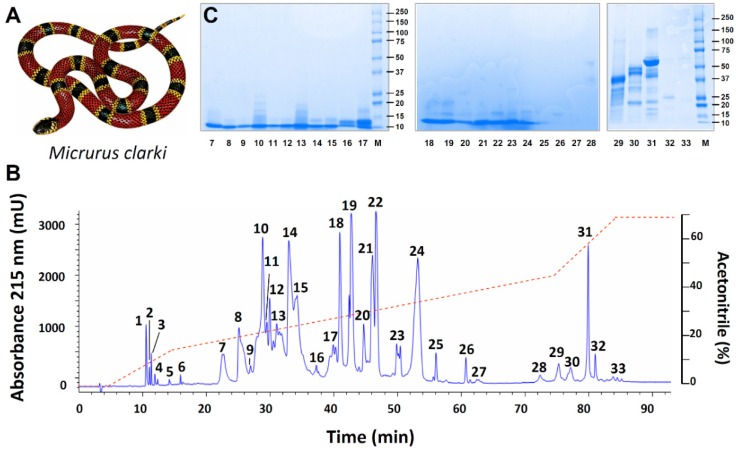
(**A**) *Micrurus clarki* and (**B**) separation of its venom (2 mg) by RP-HPLC, followed by (**C**) SDS-PAGE. Venom was fractionated on a C18 RP-HPLC column and eluted with an acetonitrile gradient (dashed line) at 1 mL/min. Fractions were further separated by SDS-PAGE under reducing conditions. Molecular weight markers (Mw) are indicated in kDa. Coomassie-stained bands were excised, in-gel digested with trypsin, and subjected to MALDI-TOF/TOF analysis for assignment to protein families, as shown in Table 1.

*Micrurus clarki* (Figure 1A) is an uncommon, slender to medially robust coral snake with a reported maximum length of 920 mm, although most adults average between 400 and 600 mm. This species has a tricolored pattern with narrow yellow bands bordering the black bands followed by single red bands, and a characteristic black head cap that extends over most of the snout and parietals. *M. clarki* has a discontinuous distribution from the Tárcoles basin in Central Pacific Costa Rica southward to the Pacific lowlands of Western Panamá, where it inhabits lowlands of the Canal Zone, to Pacific Darien, and Western Colombia. Along this distribution, *M. clarki* inhabits tropical wet forest, but also transitional tropical wet-tropical dry forest, where it is mostly found at low elevations, with some exceptions reported up to 900 m above sea level [1,6,7]. Little is known about the natural history of this coral snake. The species was named in honor of H.C. Clark, a medical doctor that directed the Gorgas Memorial Institute in Panamá, considered a pioneer in tropical medicine [8]. It is considered mainly a terrestrial and primarily nocturnal species whose natural diet is known to include the marbled swamp eel *Synbranchus marmoratus*, and colubrid snakes [6].

Our search of the mainstream literature databases did not find any information on the venom of *M. clarki*, whose general characteristics seem to be completely unknown. As part of an ongoing effort to characterize the venom proteomes of all snake species from Costa Rica [9], in the present work we report the composition, toxicity, immunological cross-recognition, and neutralization of the venom of *M. clarki* obtained from specimens collected in the Southeastern Pacific of this country. In addition, a three-finger toxin from this venom was isolated and characterized.

## 2. Results and Discussion

### 2.1. Proteomic Profile of Micrurus Clarki Venom

**Figure 2:**
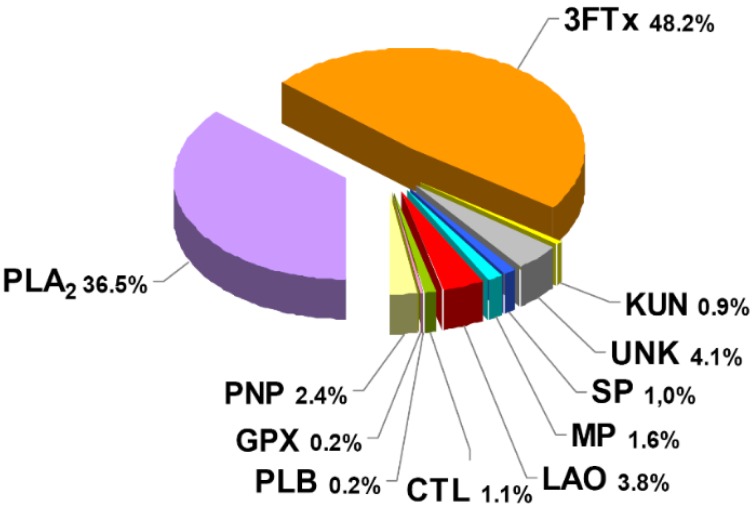
Composition of *Micrurus clarki* venom proteome according to protein families, expressed as percentages of the total protein content. 3FTx: three-finger toxin; PLA_2_: phospholipase A_2_; LAO: L-amino acid oxidase; CTL: C-type lectin/lectin-like; MP: metalloproteinase, SP: serine proteinase; KUN: Kunitz-type serine proteinase inhibitor; GPX: Glutathione peroxidase; PLB: phospholipase B; PNP: peptides and/or non-proteinaceous components; UNK: unknown/unidentified.

The venom of *M. clarki* (1A) was initially separated into 33 fractions by RP-HPLC (Figure 1B), which were further resolved into 42 protein bands after SDS-PAGE (Figure 1C). After in-gel tryptic digestion of these, followed by MALDI-TOF-TOF analysis, protein family assignments were obtained for 34 of the bands, estimated to represent 95.9% of the total venom proteins. The remaining eight bands for which no identifications were obtained (indicated as ‘‘unknown’’ in Table 1 and Figure 2), altogether add to the remaining 4.1% of the venom proteins.

Although not markedly predominant, the protein family with the highest abundance in *M. clarki* venom corresponds to three-finger toxins (3FTx), which account for almost half of the protein content (48.2%), followed by phospholipase A_2_ (PLA_2_), representing roughly one-third (36.5%).

**Figure 3:**
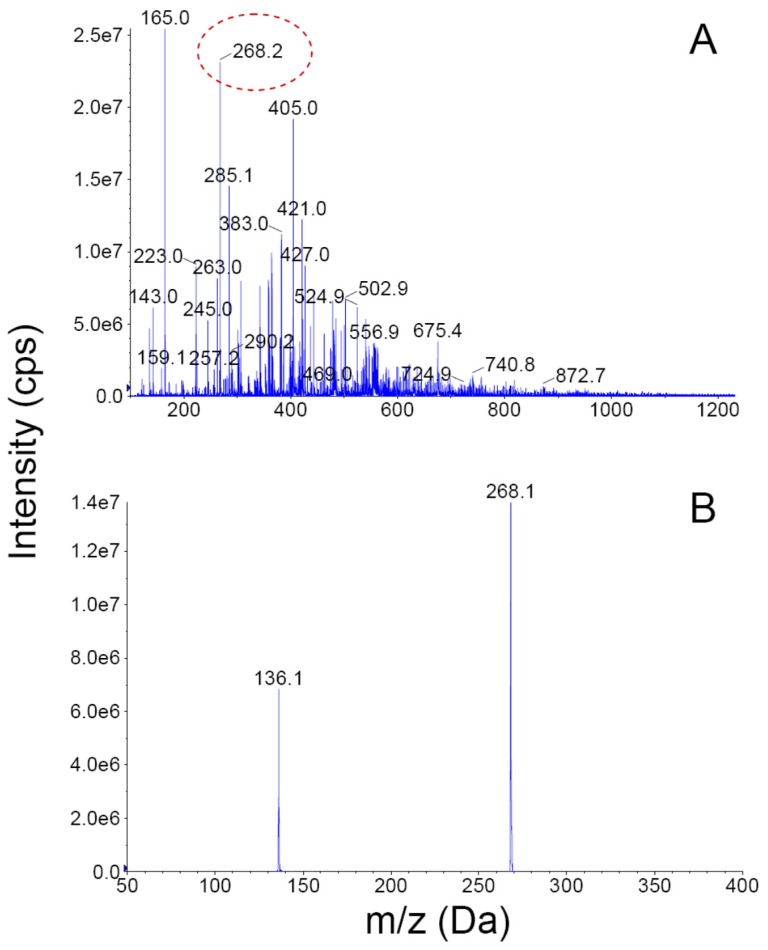
Detection of adenosine in *Micrurus clarki* venom by nESI-MS/MS. (**A**) Fraction 1 from Figure 1 was directly infused into the nano-spray ion source of a QTrap 3200 mass spectrometrer, and scanned in positive enhanced MS mode; and (**B**) the ion labeled at *m/z* 268.2 (dotted red circle in **A**) was selected for collision-induced dissociation, showing the characteristic fragment of *m/z* 136.1, which corresponds to the transition of adenosine to adenine.

In addition to its abundant 3FTxs and PLA_2_s, other proteins detected in lower proportions (within the range of 0.9%–3.8%) belong to the L-amino acid oxidase, metalloproteinase, serine proteinase, Kunitz-type serine proteinase inhibitor, and C-type lectin/lectin-like families, together with traces (~0.2%) of phospholipase B and glutathion peroxidase components (Figure 2). Altogether, proteins in this venom belong to at least nine families. As expected, the rapidly eluting peaks in the RP-HPLC separation of the venom (Figure 1B; peaks 1–6), did not show proteins by electrophoresis, and are considered to contain either small peptides or non-proteinaceous compounds (PNP; 2.4%). The most intense of these initial peaks (1–3) were analyzed by nESI-MS, and clear evidence for adenosine being present in peak 1 was obtained (Figure 3). Adenosine has been found in several venoms from *Micrurus* species, although not ubiquitously [10,11], and may have a contributing role in envenoming strategies [12], a hypothesis which still remains to be studied in detail.

### 2.2. Immunological Properties of Micrurus Clarki Venom

**Figure 4:**
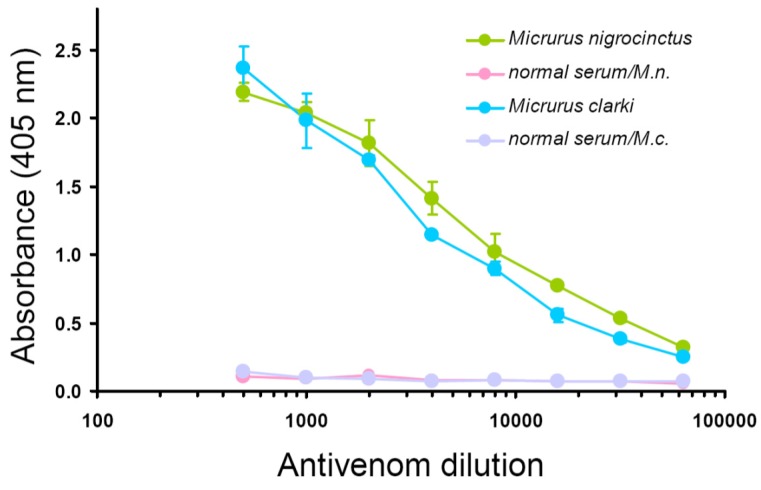
Cross-recognition of the venom of *Micrurus clarki* by an equine antivenom raised against *M. nigrocinctus* venom (SAC-ICP), as evaluated by ELISA. Crude venom from each of the two species was coated onto microplates at 1 μg/well, as described in Methods. Serial dilutions of the antivenom were added and the binding of antibodies was detected by anti-equine immunoglobulins conjugated to alkaline phosphatase, followed by color development using *p*-nitrophenylphosphate substrate. A mock antivenom prepared using the plasma of a normal, non-immunized horse, was used as a negative control for background. Each point represents mean ± SD of triplicate wells.

**Figure 5:**
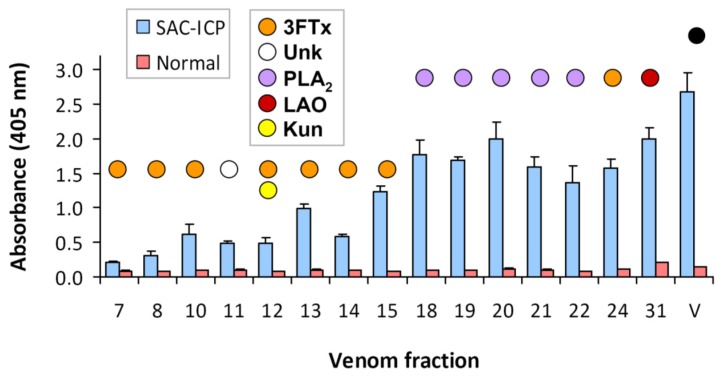
Immunorecognition of the RP-HPLC fractions of *Micrurus clarki* venom by an equine monospecific antivenom raised against *M. nigrocinctus* venom (SAC-ICP), as evaluated by ELISA. Venom fractions (0.4 μg/well) were coated onto microplates, and the binding of antibodies was detected as described in Methods, using anti-equine immunoglobulins conjugated to alkaline phosphatase, followed by color development using *p*-nitrophenylphosphate substrate. Mock antivenom prepared using the plasma of a normal, non-immunized horse, was used as a negative control for background. Each bar represents mean ± SD of triplicate wells. All bars corresponding to SAC-ICP have values that are significantly higher (*p* < 0.05; Student’s *t*-test) than controls. Colored circles above the bars indicate the protein family identified in each chromatographic fraction: three-finger toxin (3FTx), unknown (Unk), phospholipase A_2_ (PLA_2_), L-amino acid oxidase (LAO), and Kunitz-type serine protease inhibitor (Kun). The black circle represents the crude venom (V) control.

Since the antivenom used therapeutically for coral snake envenomings in Central America (SAC-ICP) is prepared by immunization of horses with the venom of a single species, *M. nigrocinctus*, it was of interest to characterize its immunological cross-recognition and cross-neutralization toward the untested venom of *M. clarki*. As shown by ELISA titration curves, this antivenom strongly cross-recognized *M. clarki* whole venom, resulting in antibody binding signals virtually as high as those obtained for the homologous (*M. nigrocinctus*) venom (Figure 4). This finding evidences the high degree of antigenic conservation between venoms of these two coral snake species. Following this result, a more detailed cross-recognition analysis was performed using the RP-HPLC-separated venom fractions of *M. clarki*, revealing additional information. First, it evidenced that the antivenom contains antibodies against all of the venom fractions tested, since the binding signals recorded were, in all cases, significantly higher than those of the negative control equine immunoglobulins (Figure 5). Second, it showed that immunorecognition of smaller proteins, such as 3FTxs or Kunitz-type components, is generally weaker in comparison to recognition of the larger PLA_2_s or LAAOs (Figure 5). The lower titers of antibodies against low molecular mass proteins may reflect differences in their immunogenicity, and/or epitope availability in the immunoassay, and is in agreement with earlier studies performed with other *Micrurus* venoms [13,14] or with venoms from other elapids [15,16].

### 2.3. Lethal Activity of Micrurus Clarki Venom and its Neutralization by Antivenom

Immunorecognition results obtained for *M. clarki* venom or its main fractions predicted that the SAC-ICP antivenom should be capable of efficiently cross-neutralizing its toxicity. Since no information was available on the lethal potency of this venom, experiments were first performed to estimate this value, indicating an intravenous median lethal dose (LD_50_) of 12.4 μg/mouse (95% confidence limits of 7.1–23.5 μg/mouse) or 0.73 μg/g body weight (0.42–1.38 μg/g). Using 4 × LD_50_ of venom (50 μg) as a challenge, the SAC-ICP antivenom indeed protected all mice from intravenous lethality when preincubated with the venom at all ratios tested (100, 200, 300, and 400 μg venom/mL antivenom). Due to the low amounts of venom available, we could not test neutralization beyond the 400 μg/mL ratio in order to estimate the median Effective Dose (ED_50_). Nevertheless, these results indicate that the neutralization potency of SAC-ICP against the lethal action of *M. clarki* venom is at least as high as that against the homologous (*M. nigrocinctus*) venom (unpublished data from the Quality Control Lab of Instituto Clodomiro Picado), in agreement with the immunological cross-recognition results (Figure 4 and Figure 5).

### 2.4. Phospholipase A_2_ and Myotoxic Activities of Micrurus Clarki Venom

**Figure 6:**
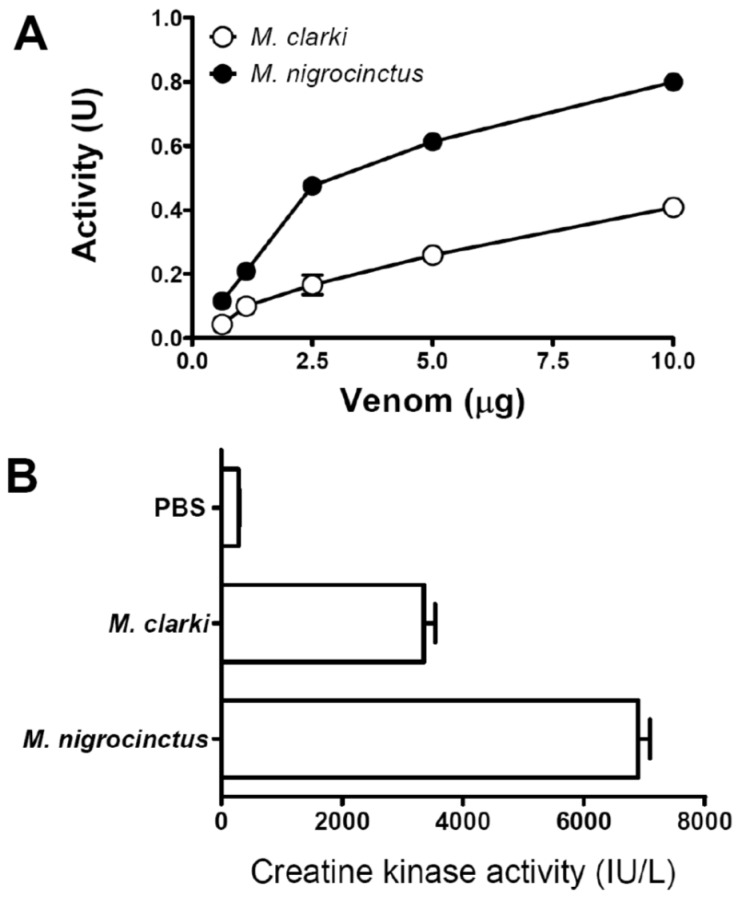
(**A**) Phospholipase A_2_ activity of the venoms of *Micrurus clarki* and *M. nigrocinctus* upon the monodisperse synthetic substrate 4-nitro-3-octanoyloxy-benzoic acid, under conditions described in Methods. Each point represents mean ± SD of triplicate assays; and (**B**) myotoxic activity of the venoms of *M. clarki* and *M. nigrocinctus* in mice. Venoms (5 μg/50 μL) or phosphate-buffered saline (PBS, 50 μL) were injected by intramuscular route and the plasma creatine kinase activity was determined 3 h later. Each bar represents mean ± SD of four mice per group.

The venom of *M. clarki* showed PLA_2_ activity upon the synthetic substrate NOBA (Figure 6A), in agreement with the presence of proteins, identified as PLA_2_s in its composition (Figure 2 and Table 1). In comparison to the venom of *M. nigrocinctus*, that of *M. clarki* presents a lower activity (Figure 6). This finding correlates well with the proportion of PLA_2_ enzymes found in the two venoms: 48.0% in *M. nigrocinctus* [13], and 36.5% in *M. clarki* (Figure 2).

On the other hand, the venom of *M. clarki* displayed a significant myotoxic activity in mice (Figure 6B). This effect was evidently less potent in comparison to that of *M. nigrocinctus* venom, under identical assay conditions (Figure 6B). This observation is also in line with the differences in PLA_2_ content in the venoms of these two species, since the myotoxicity induced by *Micrurus* venom has been related to PLA_2_ enzymes [13,14].

### 2.5. Lethality Screening of Micrurus Clarki Venom Fractions in Mice

Aiming to identify which of the venom proteins *M. clarki* contribute to its lethal effect in mice, and, therefore, predict their potential relevance to human envenomings, screenings were performed with its most abundant RP-HPLC fractions, using a challenge of 10 μg/mouse by the intravenous route. Intriguingly, fractions 7, 8, 10, 13, 14, 15, 24 (corresponding to 3FTxs), 12 (containing a 3FTx and a Kunitz-type protein), 18, or 19 (corresponding to PLA_2_s) did not induce lethality in this screening. Of all fractions tested, only fraction 21, corresponding to a PLA_2_, was individually lethal to mice. This finding is surprising, since many 3FTxs in snake venoms act as post-synaptic blockers of nicotinic acetylcholine receptors at the neuromuscular junction of mammals and/or birds, therefore being endowed with potent lethal effect [17,18,19]. The possible loss of activity by the compact, disulfide-rich 3FTxs, due to exposure to acidic acetonitrile gradients during their RP-HPLC separation seems unlikely, since a previous study showed that such procedure did not reduce the lethal potency of *Dendroaspis polylepis* (Elapidae) venom, in which 3FTxs play a predominant role [15]. Therefore, it is puzzling that lethality was observed only in a single (PLA_2_) fraction of the chromatographic profile of *M. clarki* venom, and in none of the 3FTx fractions. This finding is suggestive of possible synergisms among different venom components to induce lethality. However, the evaluation of this possibility requires more detailed studies, which could not be performed due to the scarcity of venom.

### 2.6. Characterization of Clarkitoxin-I

**Figure 7:**
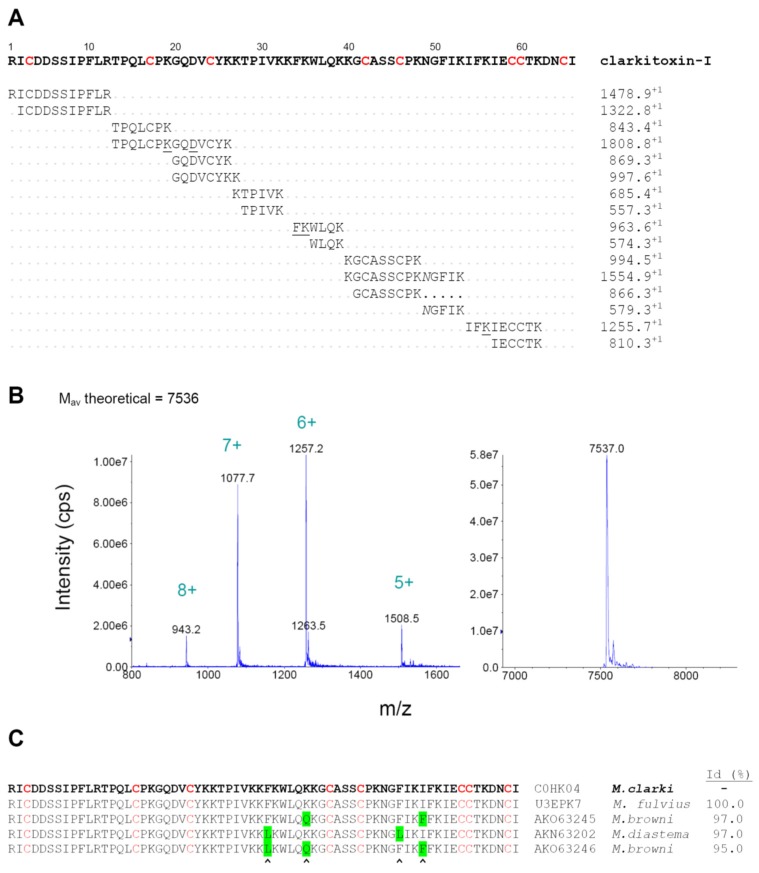
Biochemical characterization of clarkitoxin-I, a three-finger toxin isolated from the venom of *Micrurus clarki*. (**A**) Amino acid sequence of clarkitoxin-I, as determined by MALDI-TOF/TOF of tryptic peptides. The protein corresponds to peak 24 of the RP-HPLC separation of venom presented in Figure 1. *De novo*-derived sequences from the peptides and their observed monoisotopic masses are indicated below the assembled sequence. Cysteine residues (red) were carboxamidomethylated. Few residues additionally carboxamidomethylated in the tryptic digests are underlined, and asparagine residues that showed deamidation are indicated by italics. The *C*-terminal four residues (DNCI) were not observed in the analysis of tryptic peptides, but their presence is deduced on the basis of matching the isotope-averaged mass observed for the intact protein (**B**) and the theoretically calculated mass. Moreover, these four amino acids at the *C*-terminal region are conserved in the alignment of clarkitoxin-I with four homologous deduced proteins (**C**) reported in venom gland transcripts of *M. fulvius*, *M. browni*, and *M. diastema*. Amino acid positions that differ in comparison to clarkitoxin are highlighted in green, and sequence identity values are shown at the right (Id%). The sequence here reported for clarkitoxin-I will appear in the UniProt Knowledgebase under the accession number C0HK04.

Of particular interest was the most abundant venom component, peak 24, representing 16.1% of the total proteins, which was identified as a member of the 3FTx family (Table 1) but was devoid of lethal effect in mice. By performing an in-solution trypsin digestion and MALDI-TOF-TOF *de novo* sequencing of RP-HPLC-separated peptides, the complete amino acid sequence of this protein, hereby named clarkitoxin-I, was determined (Figure 7A). Despite the fact that this protein is not toxic in mice, we have coined the name ‘clarkitoxin’ because it belongs to the family of the 3FTxs, and because it might be toxic for other species, particularly natural prey species [20]. Its *C*-terminal four residues (DNCI) were not directly observed in the analysis of tryptic peptides, but their presence is deduced on the basis of (a) matching the isotope-averaged mass (Mav) observed for the intact protein of 7537 ± 1 Da (Figure 7B) with its theoretically-expected Mav of 7536 Da, according to this sequence; and (b) the absolute conservation of the DNCI sequence in four highly similar deduced sequences of venom gland transcripts or cDNAs from *M. browni* (AKO63246, AKO63245), *M. diastema* (AKN63202), and *M. fulvius* (U3EPK7) (Figure 7C). The protein sequence data reported in this paper will appear in the UniProt Knowledgebase under the accession number C0HK04. Clarkitoxin-I presents 66 amino acid residues, with the conserved pattern of eight cysteine positions and spacings that identifies it as a short-chain (type I) 3FTx, and is indeed identical to the sequence predicted by transcript U3EPK7 (cluster 10) sequenced from the venom gland transcriptome of *M. fulvius* [21], which however has not been isolated and characterized at the protein level. Three homologous sequences from *M. browni* and *M. diastema* present identities of 95%–97% in comparison to clarkitoxin-I (Figure 7C), but the next most similar protein retrieved in a BLAST search, F5CPD3 from *M. altirostris*, drops abruptly to a value of 50% identity. This large gap in similarity suggests that clarkitoxin-I, together with the closely-related variants described in *M. fulvius*, *M. diastema*, and *M. browni*, may form a group that should be phylogenetically distant from other proteins of the elapid 3FTx family, and most likely of uncharacterized functional properties (owing to the scarcity of *Micrurus* venoms).

Clarkitoxin-I was not lethal when injected by the intravenous route at 10 μg/mouse, and even the intraperitoneal injection of 30 μg into a single mouse did not cause death or evident abnormal signs. Therefore, this 3FTx protein can be considered to be devoid of significant α-neurotoxicity in mice. Toxicity to other groups of organisms such as small colubrids or swamp eels, being natural prey for coral snakes [6], was not tested and, thus, remains an open possibility. In this regard, a rational explanation for the prominent abundance of this protein in *M. clarki* venom cannot yet be proposed. Determination of the precise target specificity of this 3FTx is a pending task, which will require an adequate source of protein, such as recombinant or synthetic, due to the difficulties of obtaining venom from this coral snake species.

### 2.7. Dichotomy of Venom Types in Micrurus and the Position of Micrurus Clarki

The increasing proteomic data on *Micrurus* venoms have revealed a dichotomy between 3FTx- and PLA_2_-predominant types, which shows a correlation with phylogenetic relationships among the taxa [15]. In this regard, by containing a moderately higher proportion of 3FTxs than PLA_2_s, the venom of *M. clarki* could be grouped together with 3FTx-rich venoms such as those of *M. altirostris*, *M. corallinus*, *M. mipartitus*, *M. multifasciatus*, and *M. alleni* [15,22,23]. However, it should be noted that in the venoms of these latter coral snakes, 3FTxs represent a considerably higher proportion of the total proteins (61.1%–83.0%), and their relative PLA_2_ content, consequently, is markedly lower (8.2%–29.0%). Considering that the predominance of 3FTxs over PLA_2_s (48.2% *vs.* 36.5%, respectively) is less pronounced in the venom of *M. clarki*, its compositional pattern appears to differ from the markedly 3FTx-predominant *Micrurus* venoms described to date, and seems to represent a more ‘intermediate’ pattern. In fact, some functional and immunological characteristics of *M. clarki* venom show similarities with those observed in the group of PLA_2_-rich venoms. For example, *Micrurus* venoms from Costa Rica, which present the 3FTx-predominant expression pattern, have been found to diverge antigenically from the (PLA_2_-predominant) venom of *M. nigrocinctus*, used for the production of therapeutic antivenom in the region. As a consequence, they are weakly neutralized (*M. alleni*) or not neutralized at all (*M. mipartitus*/*M. multifasciatus*) by this antivenom [15,23]. In contrast, the present study shows that the venom of *M. clarki* clearly departs from this trend, by presenting a strong immunological cross-reactivity with the venom of *M. nigrocinctus*, which leads to an excellent cross-neutralization by its antivenom. An additional difference between *M. clarki* venom and 3FTx-predominant venoms, e.g., *M. mipartitus* from Colombia, can be pointed out from the present findings. The latter contains a highly abundant (28%) 3FTx, mipartoxin-I, which is potently lethal to mice [23,24], whereas the most abundant protein in *M. clarki* venom, clarkitoxin-I (16%), is hereby characterized as a 3FTx that lacks a lethal effect in mice. Additionally, the venom of *M. clarki* showed a considerable myotoxic effect in mice, in contrast with the 3FTx-rich venoms of *M. alleni* [11] or *M. mipartitus* [23], which do not display significant myotoxicity. Moreover, the amino acid sequence of clarkitoxin-I, its most abundant component, reveals a high conservation (95%–100% identity) in at least three other *Micrurus* species (*M. diastema*, *M. browni*, and *M. fulvius*) from the North American clade, displaying a PLA_2_-predominant pattern [15,25,26]. Although a definitive phylogenetic hypothesis for the species included in the genus *Micrurus* is still pending, a preliminary analysis of the phylogenetic positions of monadal coral snakes of Middle America based on two mitochondrial genes [27] has shown a close relationship between *M. clarki* and the *M. nigrocinctus*-*M. fulvius* clade. Altogether, the present results portray the venom of *M. clarki* as being only moderately 3FTx-predominant, displaying a more intermediate pattern between the two poles of the 3FTx/PLA_2_ dichotomy, and sharing several functional and antigenic characteristics with the PLA_2_-predominant *Micrurus* venoms.

## 3. Conclusions

To the best of our knowledge, the venom of *M. clarki* is studied here for the first time, by presenting its proteomics-based compositional profile, toxicity, and immunological recognition/neutralization properties. Protein composition indicates that this venom contains a higher proportion of 3FTxs over PLA_2_s, however not with a predominance as marked as in other coral snake venoms previously described to express the ‘3FTx-rich’ pattern. To further investigate the relationship between phylogeny and venom composition in New World elapids [15] it will be of importance to increase the number of *Micrurus* venoms characterized by means of a venomics approach, since only a minor fraction of the nearly 70 coral snake species distributed in America have had such type of analysis performed. The present study completes the venom proteomic profiling of all five coral snake species reported in Costa Rica, namely *M. nigrocinctus*, *M. mosquitensis*, *M. alleni*, *M. multifasciatus* (also described as *M. mipartitus*), and *M. clarki*, revealing a remarkable diversity in their protein composition, toxicity, and antigenic properties.

## 4. Experimental Section

### 4.1. Venom and Antivenom

Venom was obtained from three *M. clarki* adult specimens, all collected in localities of the Southeastern Pacific region of Costa Rica (ICP-078 Punta Mala, Puerto Cortés, Puntarenas, Costa Rica; ICP-079 Lagunas, Aguirre, Puntarenas, Costa Rica; and ICP-806 Platanillo, Pérez Zeledón, San José, Costa Rica) and kept at the Serpentarium of Instituto Clodomiro Picado, University of Costa Rica. Venom samples were pooled, lyophilized, and stored at −20 °C until analysis. A total of ~15 mg of venom was obtained. For the purpose of studying immunological cross-recognition and neutralization of this venom, the monospecific equine anti-coral snake antivenom against *M. nigrocinctus*, produced at Instituto Clodomiro Picado (SAC-ICP; batch 520, expiry date March 2016) was used.

### 4.2. Proteomic Profiling of the Venom

The ‘‘snake venomics’’ analytical strategy [28] was followed, as previously described [29]. In brief, the venom was decomplexated by a RP-HPLC step, followed by SDS-PAGE of each of the obtained fractions. Protein bands were excised and in-gel digested with trypsin after reduction and alkylation. The obtained peptides were submitted to MALDI-TOF/TOF mass spectrometry on a Proteomics Analyzer 4800 Plus (Applied Biosystems, Foster City, CA, USA) instrument for assignment to known protein families by similarity with sequences contained in the UniProt/SwissProt database (Serpentes, January 2016), using the ProteinPilot^®^ software (ProteinPilot Software v.4.0, 2010) and the Paragon^®^ algorithm (ABsciex, Redwood City, CA, USA), at a confidence level of ≥95%.

### 4.3. Quantitative Estimation of the Protein Family Composition of the Venom

The relative abundance of each protein (% of total venom proteins; [29]) was estimated by integration of the RP-HPLC peak signals at 215 nm, using Chem Station^®^ B.04.01 software (Agilent, Santa Clara, CA, USA, 2009). For HPLC peaks presenting more than one SDS-PAGE band, percentage distribution was assigned by densitometry, using a ChemiDoc^®^ recorder and Image Lab^®^ v.2.0 software (Bio-Rad, Hercules, CA, USA).

### 4.4. Immunological Cross-Recognition of Venom and its RP-HPLC Fractions

The ability of equine antivenom raised against *M. nigrocinctus* venom (SAC-ICP) to cross-recognize whole *M. clarki* venom, or its RP-HPLC fractions, was assessed by ELISA [14]. Crude venoms of *M. clarki* and *M. nigrocinctus* were dissolved in 0.1 M Tris, 0.15 M NaCl, pH 9.0 buffer, and coated onto microplates (Nunc) at 1 µg/100 µL/well, overnight at 4 °C. Plates were washed five times with PBS (0.12 M NaCl, 40 mM sodium phosphate, pH 7.2), blocked for 60 min with 100 µL/well of PBS containing 2% bovine serum albumin (BSA), and decanted. Then, 100 µL of antivenom (serially diluted from 1:500 to 1:64,000) were added and incubated for 2 h. An identical preparation of immunoglobulins obtained from a non-immunized horse was used as a negative control. Plates were washed five times with PBS, followed by the addition of 100 µL of anti-equine immunoglobulins-alkaline phosphatase conjugate (Sigma), diluted 1:4000 in PBS-BSA, and incubated for 2 h. After five washings with PBS, color was developed with 1 mg/mL *p*-nitrophenylphosphate in diethanolamine buffer, pH 9.8, and absorbances were recorded at 405 nm. All samples were assayed in triplicate wells. A similar ELISA procedure was used to assess the recognition of RP-HPLC fractions of *M. clarki* venom by the SAC-ICP antivenom. In this case, microplates were coated with 0.4 µg/100 µL/well of each fraction and the binding of equine antibodies was tested at an antivenom dilution of 1:1000, in PBS-BSA, as described above.

### 4.5. Mass Determination of Clarkitoxin-I by Electrospray Mass Spectrometry

The protein from peak 24 (2 µg) of the RP-HPLC separation of *M. clarki* venom was diluted in 10 µL of 50% acetonitrile containing 0.1% formic acid, and loaded into a metal-coated glass capillary (Proxeon) for direct infusion into a QTrap3200 mass spectrometer (Applied Biosystems, Foster City, CA, USA) using a nano-electrospray ionization source operating at 1200 V. The sample was analyzed in the positive enhanced multi-charge mode, and the ion series was deconvoluted with the aid of the Analyst v.1.5 software (ABsciex, Redwood City, CA, USA, 2008) to obtain the isotope-averaged molecular mass (M_av_) of the protein.

### 4.6. Amino Acid Sequencing of Clarkitoxin-I by Tandem Mass Spectrometry

The protein from peak 24 (75 µg) of the RP-HPLC separation of *M. clarki* venom was dissolved in ammonium bicarbonate (50 mM), reduced with dithiothreitol (10 mM) for 30 min at 56 °C, and alkylated with iodacetamide (50 mM) for 20 min in the dark. Sequencing grade trypsin (1.5 µg) was added and incubated for 18 h at 37°C. The resulting peptide mixture was separated by RP-HPLC on a C18 column (2.1 × 150 mm, 5 µm particle diameter) eluted at 300 µL/min with a gradient from 0.1% trifluoroacetic acid (TFA) in water (solvent A) to acetonitrile/0.1% TFA (solvent B) as follows: 10 min at 0% B, 5 min to 12% B, 30 min to 30% B, 5 min to 70% B, and 10 min at 70% B. Peptides were collected manually, dried by vacuum centrifugation, and redissolved in 20 µL of 50% acetonitrile containing 0.1% TFA, followed by MALDI-TOF/TOF analysis as previously described [29].

### 4.7. Venom Lethality and its Neutralization by Antivenom

Venom was dissolved in PBS, and various doses were injected by the intravenous route into groups of four CD-1 mice of 16–18 g of body weight, of either sex. Deaths occurring during the next 48 h were recorded and the median lethal dose (LD_50_) for the venom was calculated by probits [30].

The ability of the equine antivenom against *M. nigrocinctus* (SAC-ICP) to neutralize the lethal activity of *M. clarki* venom was assessed by a preincubation-type approach. Groups of four mice (16–18 g) were injected intravenously with 100 µL of a solution that contained 50 µg of venom (equivalent to 4 × LD_50_), which had been previously mixed and incubated for 30 min at 37 °C with antivenom in order to attain various venom/antivenom ratios: 100, 200, 300, and 400 µg of venom/mL of antivenom. A control group of mice received the same dose of venom, incubated with PBS instead of antivenom. Deaths occurring during the following 48 h were recorded in each group. All *in vivo* assays followed guidelines by the Institutional Committee on the Use and Care of Laboratory Animals (CICUA) at the University of Costa Rica (CICUA 041-15, 21 October 2015).

### 4.8. Phospholipase A_2_ and Myotoxic Activities of Micrurus Clarki Venom

The PLA_2_ activity of *M. clarki* venom was tested on the monodisperse synthetic substrate 4-nitro-3-octanoyloxy-benzoic acid (4-NOBA) as described [14]. Variable amounts of venoms (in 25 μL of 10 mM Tris, 10 mM CaCl2, 100 mM NaCl, pH 8.0 buffer) were added to microplate wells (in triplicates), mixed with 25 μL of substrate (1 mg/mL in acetonitrile) and 250 μL of the same buffer. After incubation for 60 min at 37 °C, absorbances were recorded at 405 nm in a microplate reader and activity was expressed as the absorbance change in comparison to the substrate alone. For comparative purposes, the venom of *M. nigrocinctus* was run in parallel in this assay, under identical conditions.

The myotoxic activity of *M. clarki* venom was tested in mice (*n* = 4), which received an intramuscular injection of 5 μg in the gastrocnemius, in a volume of 50 μL of PBS. A control group of mice received only PBS. The venom of *M. nigrocinctus* was injected in another group of mice, under identical conditions, for comparison. Three hours after injection, all animals were bled from the tip of the tail, and the activity of creatine kinase in their plasma was determined using a UV kinetic-based enzymatic assay (CK-Nac, Biocon, Brea, CA, USA), as described [14].

### 4.9. Lethality Screening of Venom Fractions

The main venom fractions obtained by the RP-HPLC separation were screened for lethal activity in mice, by injecting 10 µg/100 µL by the intravenous route and recording deaths up to 48 h, using two mice per fraction. In addition, clarkitoxin-I was injected at a dose of 30 µg in one mouse, by the intraperitoneal route.

## Figures and Tables

**Table 1:** Assignment of the RP-HPLC/SDS-PAGE separated fractions of *Micrurus clarki* venom to protein families by MALDI-TOF-TOF of selected peptide ions from in-gel trypsin-digested protein bands.

Peak	%	Mass, kDa ▼ (Da)	Peptide Ion		MS/MS-Derived Sequence *	Conf (%)	Sc **	Protein Family; ~ Related Protein and Species ***
			*m/z*	*z*				
1	1.3	-	-	-	-	-	-	PNP; adenosine
2–6	1.1	-	-	-	-	-	-	PNP
7	3.2	10 ▼ (6757)	1707.0	1	FSPGIQTSQTCPAGQK	99.0	11	3FTx; ~U3FVH8, 3FTx 4a *Micrurus fulvius*
8	5.9	9 ▼ (6638, 7276)	976.5	1	MFIRTHR	76.6	5	3FTx; ~P01414 , toxin FS-2 *Dendroaspis polylepis*
			1322.7	1	ICDDSSIPFLR	99.0	7	3FTx; ~U3EPK7, 3FTx 6 *Micrurus fulvius*
9	0.2	9 ▼	1314.7	1	FYFAYQCTSK	man	man	3FTx; ~C6JUP1, 3FTx *Micrurus corallinus*
10	8.0	9 ▼ (6361, 6451)	2663.2	1	DGFYSVTCTEKENLCFTMFSAR	99.0	11	3FTx; ~C6JUP4, 3FTx *Micrurus corallinus*
			1375.6	1	ENLCFTMFSAR	99.0	11	
11	0.4	9 ▼	-	-	-	-	-	Unknown
12	1.8	9 ▼ (7045, 7140, 7198)	1661.8	1	GAYNVCCSTDLCNK	99.0	15	3FTx; ~U3FAE1, 3FTx 3a *Micrurus fulvius*
			1374.7	1	KCLEFIYGGCQ	99.0	12	Kunitz; ~Q6ITB9, mulgin-3 *Pseudechis australis*
13	3.6	9 ▼ (7071, 7201)	1786.9	1	VCYTIFLVGPSYP^py^EK	93.5	6	3FTx; ~AKN63197, 3FTx *Micrurus diastema*
14	6.9	9 ▼ (6845)	1687.8	1	GPYNVCCSTDLCNK	99	7	3FTx; ~B3EWF8, mipartoxin-I *Micrurus mipartitus*
			1310.7	1	AIEFGCAASCPK	99	12	3FTx; ~AKN63198, 3FTx DK, *Micrurus diastema*
15a	0.4	12 ▼	-	-	-	-	-	unknown
15b	2.5	9 ▼ (6843, 7311)	1314.7	1	FYFAYQCTSK	man	man	3FTx; ~C6JUP1, 3FTx *Micrurus corallinus*
16a	0.3	12 ▼	-	-	-	-	-	unknown
16b	0.2	10 ▼	1373.6	1	CKDFVCNCDR	99.0	7	PLA_2_; ~U3FYP8, PLA_2_ 13 *Micrurus fulvius*
17a	1.7	13 ▼	1373.6	1	CKDFVCNCDR	99.0	14	PLA_2_; ~U3FYP8, PLA_2_ 13 *Micrurus fulvius*
			1085.4	1	DFVCNCDR	96.8	6	
17b	1.2	9 ▼	-	-	-	-	-	unknown
18	6.6	13 ▼ (13353)	2969.3	1	HWVSFTNYGCYCGYGGSGTPVDELDK	99.0	12	PLA_2_; ~P81167, nigroxin B *Micrurus nigrocinctus*
			1373.5	1	CKDFVCNCDR	99.0	12	PLA_2_; ~U3FYP8, PLA_2_ 13 *Micrurus fulvius*
			2983.3	1	HWLSFTNYGCYCGYGGSGTPVDELDK	98.5	6	
19	11.3	13 ▼ (13477)	1373.6	1	CKDFVCNCDR	99.0	9	PLA_2_; ~U3FYN8, PLA_2_ 9 *Micrurus fulvius*
20	1.8	13 ▼	2855.3	1	SAWDFTNYGCYCGAGGSGTPVDELDR	99.0	15	PLA_2_; ~Q8AXW7, PLA_2_ *Micrurus corallinus*
21	5.3	12 ▼ (13331)	2841.3	1	AWDYNN^da^YGCYCGQGGSGTPVDDLDR	99	12	PLA_2_; ~R4G7M2, Sut-22 *Suta fasciata*
			1335.7	1	VHDDCYAAAEK	99	18	PLA_2_; ~ U3FYP1, PLA_2_ 3b *Micrurus fulvius*
			1827.8	1	CCQVHDNCYNEAEK	99	22	PLA_2_; ~ A4FS04, natratoxin, *Naja atra*
22a	2.4	20 ▼	1278.6	1	VHDDCYAAAEK	99.0	7	PLA_2_; ~U3FYP1, PLA_2_ 3b *Micrurus fulvius*
22b	4.5	13 ▼	1373.5	1	CKDFVCNCDR	99.0	8	PLA_2_; ~U3FYP8, PLA_2_ 13 *Micrurus fulvius*
			2883.2	1	S^f^°AWDFTNYGCYCGAGGSGTPVDELDR	99.0	15	PLA_2_; ~Q8AXW7, PLA_2_ *Micrurus corallinus*
23a	0.6	13 ▼	1278.5	1	VHDDCYAAAEK	99.0	8	PLA_2_; ~U3FYP1, PLA_2_ 3b *Micrurus fulvius*
23b	2.1	11 ▼	2883.3	1	PW^ky^IGYVNYGCYCGAGGSGTPVDELDR	99.0	16	PLA_2_; ~P00606, PLA_2_ *Bungarus multicinctus*
24	16.1	10 ▼ (7537)	1478.7	1	RICDDSSIPFLR	99	12	3FTx; ~U3EPK7, 3FTx 10a *Micrurus fulvius*
			1322.6	1	ICDDSSIPFLR	99	15	
			1108.6	1	KGCASSCPKN	99	13	
25	0.9	10 ▼	1322.6	1	ICDDSSIPFLR	99.0	9	3FTx; ~U3EPK7, 3FTx 6 *Micrurus fulvius*
26	1.1	22 ▼	1183.7	1	NVWIGLNDPR	man	man	CTL; ~ACC67944, mannose-binding 1 *Pseudechis porphyriacus*
27	0.1	-	-	-	-	-	-	unknown
28a	0.2	38 ▼	1054.5	1	TYWHYER	99.0	11	MP; ~U3FWL3, MTP4 *Micrurus fulvius*
			1130.6	1	EVFDGHTIGR	man	man	MP; ~P85314, MP *Bothrops moojeni*
28b	0.2	29 ▼	1297.6	1	SAECPTDSFQR	99.0	7	MP: ~R4G7J1, MP-Hop-13 *Hoplocephalus bungaroides*
29a	0.6	38 ▼	-	-	-	-	-	unknown
29b	0.2	28 ▼	1011.6	1	FYVVVDNR	90.9	9	MP; ~ABQ01135, stephensease-1 *Hoplocephalus. stephensii*
29c	0.2	25 ▼	1011.6	1	FYVVVDNR	man	man	MP; ~ABQ01135, stephensease-1 *Hoplocephalus. stephensii*
30a	0.4	51 ▼	995.6	1	F^dh^YVVVDNR	man	man	MP; ~ABQ01135, stephensease-1 *Hoplocephalus. stephensii*
30b	0.4	46 ▼	995.6	1	F^dh^YVVVDNR	man	man	MP; ~ABQ01135, stephensease-1 *Hoplocephalus. stephensii*
30c	0.1	29 ▼	2171.1	1	GDSGGPLICNGQIQGIVSWGR	99.0	7	SP; ~Q5MCS0, harobin *Hydrophis hardwickii*
30d	0.2	20 ▼	1329.7	1	IHDIKWNFEK	99.0	13	GPX; ~XP_013914274, glutathione peroxidase 3 *Thamnophis sirtalis*
31a	2.2	61 ▼	1637.8	1	NDLEGWHVNLGPMR	99.0	23	LAO; ~U3FYQ2, LAAO 1a *Micrurus fulvius*
			1131.5	1	SDDIFSYER	99.0	13	-
			1576.9	1	IQDNTENVRVAYR^am^	99.0	13	
			1484.7	1	EADYEEFLEIAR	99.0	19	
31b	0.5	50 ▼	1963.1	1	TSGDIVINDLSLIHQLPK	99.0	7	LAO; ~U3FYQ2, LAAO 1a *Micrurus fulvius*
			2275.1	1	IHFAGEYTANDHGWIDSTIK	99.0	8	-
			1484.7	1	EADYEEFLEIAR	99.0	9	-
			1310.7	1	RFDEIVGGM°^x^DR	99.0	10	LAO; ~A0A0A1WCY6, B variant *Echis coloratus*
			1460.8	1	QVVPESLFAWER	95.0	7	PLB: ~V8ND68, PLB-like 1 *Ophiophagus hannah*
31c	1.1	42 ▼	1484.7	1	EADYEEFLEIAR	99.0	13	LAO; ~U3FYQ2, LAAO 1a *Micrurus fulvius*
			1963.0	1	TSGDIVINDLSLIHQLPK	99.0	17	
			2275.0	1	IHFAGEYTANDHGWIDSTIK	99.0	20	
			1131.5	1	SDDIFSYER	99.0	11	
			1576.8	1	IQDNTENVRVAYR^am^	99.0	12	
31d	0.7	20 ▼	-	-	-	-	-	unknown
31e	0.2	17 ▼	1589.8	1	NDLEGWHVNLGPM^dt^R	99.0	9	LAO; ~U3FYQ2, LAAO 1a *Micrurus fulvius*
			1466.7	1	E^py^ADYEEFLEIAR	99.0	11	
			1833.8	1	EFVQEDENAWYYIK	99.0	19	
			1637.8	1	NDLEGWHVNLGPMR	99.0	12	
			1484.7	1	EADYEEFLEIAR	99.0	17	
31f	0.4	14 ▼	-	-	-	-	-	unknown
32	0.9	23 ▼	1152.6	1	LPFYADWIK	99.0	12	SP; ~U3FBP8, prostasin-like protein *Micrurus fulvius*

▼ reduced SDS-PAGE apparent mass, in kDa. Selected peaks were analyzed by mass spectrometry, and their masses (Da) are shown in parentheses; * Cysteine residues are carbamidomethylated. Possible, although unconfirmed/ambiguous amino acid modifications, suggested by the automated identification software are shown in superscript, with the following abbreviations: ox: oxidized; da: deamidated; py: pyroglutamic; fo: formyl; dh: dehydrated; am: amidated; ky: hydroxykynurenin; dt: dethiomethyl; ** Confidence (Conf) and score (Sc) values are calculated by the Paragon^®^ algorithm of ProteinPilot^®^ v.4.0. Few spectra that were manually resolved are indicated by ‘‘man’‘; *** Abbreviations: PNP: peptide or non-proteic; 3FTx: three-finger toxin; PLA_2_: phospholipase A_2_; Kunitz: Kunitz-type serine proteinase inhibitor; CTL: C-type lectin/lectin-like; MP: metalloproteinase; SP: serine proteinase; LAO: L-amino acid oxidase; PLB: phospholibase B; GPX: glutathione peroxidase.
